# Molecular Characterisation of Vancomycin-Resistant *Enterococcus faecium* Isolates Belonging to the Lineage ST117/CT24 Causing Hospital Outbreaks

**DOI:** 10.3389/fmicb.2021.728356

**Published:** 2021-09-27

**Authors:** Paola Lisotto, Natacha Couto, Sigrid Rosema, Mariëtte Lokate, Xuewei Zhou, Erik Bathoorn, Hermie J. M. Harmsen, Alexander W. Friedrich, John W. A. Rossen, Monika A. Chlebowicz-Fliss

**Affiliations:** ^1^ Department of Medical Microbiology and Infection Prevention, University of Groningen, University Medical Center Groningen, Groningen, Netherlands; ^2^ The Milner Centre for Evolution, Department of Biology and Biochemistry, University of Bath, Bath, United Kingdom; ^3^ Department of Pathology, University of Utah School of Medicine, Salt Lake City, UT, United States; ^4^ IDbyDNA Inc., Salt Lake City, UT, United States

**Keywords:** vancomycin-resistant, *Enterococcus faecium*, outbreak, mobile genetic elements, typing, cgMLST

## Abstract

**Background:** Vancomycin-resistant *Enterococcus faecium* (VREfm) is a successful nosocomial pathogen. The current molecular method recommended in the Netherlands for VREfm typing is based on core genome Multilocus sequence typing (cgMLST), however, the rapid emergence of specific VREfm lineages challenges distinguishing outbreak isolates solely based on their core genome. Here, we explored if a detailed molecular characterisation of mobile genetic elements (MGEs) and accessory genes could support and expand the current molecular typing of VREfm isolates sharing the same genetic background, enhancing the discriminatory power of the analysis.

**Materials/Methods:** The genomes of 39 VREfm and three vancomycin-susceptible *E. faecium* (VSEfm) isolates belonging to ST117/CT24, as assessed by cgMLST, were retrospectively analysed. The isolates were collected from patients and environmental samples from 2011 to 2017, and their genomes were analysed using short-read sequencing. Pangenome analysis was performed on *de novo* assemblies, which were also screened for known predicted virulence factors, antimicrobial resistance genes, bacteriocins, and prophages. Two representative isolates were also sequenced using long-read sequencing, which allowed a detailed analysis of their plasmid content.

**Results:** The cgMLST analysis showed that the isolates were closely related, with a minimal allelic difference of 10 between each cluster’s closest related isolates. The vanB-carrying transposon *Tn*1549 was present in all VREfm isolates. However, in our data, we observed independent acquisitions of this transposon. The pangenome analysis revealed differences in the accessory genes related to prophages and bacteriocins content, whilst a similar profile was observed for known predicted virulence and resistance genes.

**Conclusion:** In the case of closely related isolates sharing a similar genetic background, a detailed analysis of MGEs and the integration point of the *vanB*-carrying transposon allow to increase the discriminatory power compared to the use of cgMLST alone. Thus, enabling the identification of epidemiological links amongst hospitalised patients.

## Introduction

Enterococci are Gram-positive cocci commonly present in the human and animal gastrointestinal tract. Amongst them, *Enterococcus faecium* has been increasingly recognised as one of the leading causes of healthcare-associated infections (HAIs) due to its intrinsic and acquired resistance to several antibiotics, such as ampicillin, gentamicin, and vancomycin (Arias and Murray, 2012; García-Solache and Rice, 2019). Multidrug resistant *E. faecium* strains represent a threat for patients with a compromised immune system making them particularly susceptible to infections. Hence, in a joint effort to fight nosocomial infections, health agencies included *E. faecium* in the list of ESKAPE pathogens for which is urgently required the development of novel therapeutics drugs (Rice, 2008). Vancomycin-resistant *E. faecium* (VREfm) can arise from HA-vancomycin-susceptible *E. faecium* (VSEfm) by acquiring the *vanA* and/or *vanB* operons present in enteric anaerobic bacteria (Stinear et al., 2001; Donskey, 2004; Domingo et al., 2005; Howden et al., 2013). Our hospital mainly encounters the *vanB* positive VREfm (Zhou et al., 2018), where the *vanB* gene is located on the conjugative transposon *Tn*1549, usually integrated into the bacterial chromosome (Bender et al., 2016; Freitas et al., 2016). On the contrary, in *vanA* positive *E. faecium*, the transposon is generally associated with *Tn*1546 located on plasmids (Novais et al., 2008).

The high genome plasticity that characterises hospital-associated *E. faecium*, also referred to as clade A1, is an important factor contributing to their acquisition of mobile genetic elements (MGEs; Lebreton et al., 2013). This population not only is resistant to multiple antibiotics, but it also holds a repertoire of virulence factors that, together with the increasing tolerance towards disinfectants and higher tenacity to survive on environmental surfaces, makes VREfm a successful nosocomial pathogen (Werner et al., 2013; Gao et al., 2018; Pidot et al., 2018; García-Solache and Rice, 2019; Gorrie et al., 2019; de Maat et al., 2020).

The application of next generation sequencing (NGS) in clinical microbiology and infection prevention has proved successful for outbreak investigation (Deurenberg et al., 2017). In this scenario, whole-genome sequencing (WGS) provides high resolution regarding the relatedness of the isolates. It allows for a detailed analysis of the MGEs content that can point to a possible epidemiological link amongst the patients (Deurenberg et al., 2017; Raven et al., 2017; Pinholt et al., 2019). However powerful, its discriminatory power is not fully deployed by core genome Multilocus sequence typing (cgMLST), currently the recommended molecular typing method of VREfm in the Netherlands. This method is based on a gene-by-gene approach where a well-established set of core genes, 1,423 for *E. faecium*, are compared amongst the isolates and differences are then translated into allelic distances (de Been et al., 2015). By cgMLST, very closely related genomes are then grouped to form a Complex Type (CT). Because this method is based on comparing a well-established set of core genes, it only allows for clonal spread investigation. In contrast, horizontal gene transfer of MGEs, such as those carrying the Van operon, could be herewith missed (Raven et al., 2017). Therefore, considering the high genomic plasticity characterising *E. faecium*, additional investigation of accessory genes and plasmids could provide the required discrimination (Schürch et al., 2018).

Here, we applied WGS to characterise and compare *vanB*-carrying VREfm isolates belonging to ST117/CT24 circulating in the University Medical Center Groningen (UMCG) between 2011 and 2017, causing two major outbreaks. We aim to analyse the genetic relatedness amongst isolates involved in the same outbreaks and, by including VSEfm and non-outbreak VREfm isolates of the same genetic background, investigate the relative contribution of the MGEs in distinguishing outbreaks from each other.

## Materials and Methods

### Study Settings and Bacterial Isolates

This study retrospectively analysed 39 VREfm and three VSEfm strains isolated at UMCG between 2011 and 2017 solely belonging to the ST117/CT24-*vanB* clone as assessed by cgMLST typing. Upon admission in our hospital, rectal swab screening for VRE is performed as described before (Zhou et al., 2018) on: (i) patients who were admitted to a hospital abroad in the last year; (ii) patients transferred from another hospital in the Netherlands; (iii) patients admitted to the intensive care and haematology wards; (iv) children adopted from abroad. VRE positive patients are treated in contact isolation and, if nosocomial acquisition is suspected, screening of contact patients is performed. These measures are lifted if at least five negative rectal swabs are obtained. In general, suspected VRE colonies were confirmed by MALDI-TOF Mass Spectrometry (Bruker), and antibiotic susceptibility testing was conducted as part of the routine screening procedures by VITEK®2 using the AST card P-586 (bioMérieux). All first VRE isolates of individual cases were sequenced as described below. In the time span analysed in this study, two outbreaks caused by this specific lineage took place, one in 2014 and one in 2017.

From the 2014 outbreak, 12 isolates were included, of which 10 have been described previously by Zhou et al. (2018). Only three isolates caused infections, whilst the other nine were isolated during preventive screening procedures from rectal swabs. Twenty-one isolates were collected during the outbreak in 2017 from rectal swabs (16), faeces (1), and environmental samples (4). Six isolates that belonged to several sporadic VREfm carriages and/or infection cases between 2011 and 2015 have been retrospectively assessed by WGS, which has been implemented in our laboratory for outbreak investigation since 2014. Finally, three VSEfm isolates were collected in 2016 from pus, pleural fluid, and rectal swabs. Finally, the genome of a fully sequenced *E. faecium* strain from Spain (Tedim et al., 2017) belonging to ST117/CT24 was used as an external reference and was referred to here as E1 strain. In total, 43 isolates were included in this study.

### WGS and Isolates Characterisation

Bacterial isolates were grown overnight on blood agar plates at 37°C. Genomic DNA was extracted using the Ultraclean Microbial DNA Isolation kit (MO BIO Laboratories, Carlsbad, CA, United States) according to the manufacturer’s instructions. DNA concentration and purity were measured by the Qubit dsDNA HS assay kit (Life Technologies, Carlsbad, CA, United States). Preparation of the DNA libraries was performed with the Nextera XT v2 kit (Illumina, San Diego, CA, United States) and sequenced in a MiSeq platform (Illumina). Trimming and *de novo* assemblies were performed in CLC Genomics Workbench v12 (QIAGEN, Hilden, Germany). Two isolates of interest, samples A12 and B11, and single representatives of the outbreaks in 2014 and 2017, were also sequenced using the MinION (Oxford Nanopore Technologies, Oxford, United Kingdom). For these two strains, base calling was performed using Albacore (v1.2.2). Data quality was analysed through Poretools (v0.6.0; Loman and Quinlan, 2014). Hybrid assemblies were performed using Unicycler (v0.4.1; Wick et al., 2017). Assembly statistics are provided in Supplementary Table 1.

Multilocus sequence typing Sequence Types (STs) and cgMLST Complex Types (CTs) were extracted from the assembled genomes using Ridom SeqSphere+ (v5.1.0; Ridom GmbH, Münster, Germany) and the *E. faecium* curated scheme published previously (de Been et al., 2015). The *vanB*-carrying transposons were identified by BLASTn comparisons of *de novo* and hybrid assemblies with the reference sequence of *Tn*1549 (GenBank AF192329.1). Detailed analysis of the integration points was performed by BLASTn comparison with the E1 strain using Artemis Comparison Tool (ACT; Carver et al., 2005) and Artemis (Carver et al., 2012). IS finder (Kichenaradja et al., 2010) was used to investigate the presence of insertion sequences in the *vanB*-carrying transposons.

The genomes were characterised *in silico* using the online tools VirulenceFinder (v2.0; Joensen et al., 2014) and ResFinder (v3.2; Zankari et al., 2012). Additionally, the genomes were investigated for the presence of bacteriocins using BAGEL4 (van Heel et al., 2018) and prophages using PHASTER (Arndt et al., 2016). The predicted prophage regions were manually extracted using Artemis (Carver et al., 2012), and multiple BLASTn analyses were performed to compare the isolates. Finally, BRIG (Alikhan et al., 2011) was used for data visualisation. Plasmid analysis was performed on the hybrid assemblies, first analysed with mlplasmids (Arredondo-Alonso et al., 2018) to predict plasmid- and chromosome-derived sequences. Afterwards, multiple BLASTn analyses were performed to compare the predicted plasmid sequences, and the results were visualised with the R package Circlize (Gu et al., 2014). Abricate (https://github.com/tseemann/abricate, version 1.0.1) was used to query (> 80% identity and > 60% coverage) the predicted plasmid sequences vs. a curated database of known relaxases proteins from *Enterococcus* (Clewell et al., 2014), PlasmidFinder database (Carattoli et al., 2014) was used to identify replication initiator proteins (RIPs). The pangenome analysis was performed using Roary (Page et al., 2015).

## Results

In this study, WGS was applied to characterise and compare VREfm sharing the same genetic background (ST117/CT24) and isolated between 2011 and 2017 from hospitalised patients and environmental samples. We analysed the genetic relatedness amongst isolates, including VSEfm isolates, and characterised MGEs and accessory genes that could potentially distinguish them. Detailed information on the investigated isolates is presented in Table 1 and Supplementary Figure 1.

**Table 1:** Epidemiological and molecular data of the isolates included in this study.

Sample	cgMLST	Vancomycin	Source	Date	Insertion sites^*^	Location *Tn*1549
A1	ST117/CT24	VRE	rectal swab	8-3-2014	BO233_RS04895	Chromosome
A2	ST117/CT24	VRE	rectal swab	28-5-2014	BO233_RS04895	Chromosome
A3	ST117/CT24	VRE	sputum	28-5-2014	BO233_RS04895	Chromosome
A5	ST117/CT24	VRE	rectal swab	16-4-2014	BO233_RS04895	Chromosome
A6	ST117/CT24	VRE	rectal swab	16-4-2014	BO233_RS04895	Chromosome
A7	ST117/CT24	VRE	rectal swab	21-4-2014	BO233_RS04895	Chromosome
A8	ST117/CT24	VRE	rectal swab	24-4-2014	BO233_RS04895	Chromosome
A10	ST117/CT24	VRE	rectal swab	1-5-2014	BO233_RS04895	Chromosome
A11	ST117/CT24	VRE	rectal swab	5-5-2014	BO233_RS04895	Chromosome
A12	ST117/CT24	VRE	rectal swab	2-4-2014	BO233_RS04895	Chromosome
A53	ST117/CT24	VRE	bile fluid	14-4-2014	BO233_RS04895	Chromosome
A54	ST117/CT24	VRE	blood	1-5-2014	BO233_RS04895	Chromosome
B1	ST117/CT24	VRE	mattress	28-1-2017	BO233_RS09450	Chromosome
B2	ST117/CT24	VRE	rectal swab	1-2-2017	BO233_RS09450	Chromosome
B3	ST117/CT24	VRE	faces	11-1-2017	BO233_RS09450	Chromosome
B4	ST117/CT24	VRE	bed/mattress	30-1-2017	BO233_RS09450	Chromosome
B5	ST117/CT24	VRE	bed/mattress	30-1-2017	BO233_RS09450	Chromosome
B7	ST117/CT24	VRE	bed/mattress	30-1-2017	BO233_RS09450	Chromosome
B8	ST117/CT24	VRE	rectal swab	13-1-2017	BO233_RS09450	Chromosome
B9	ST117/CT24	VRE	rectal swab	26-1-2017	BO233_RS09450	Chromosome
B10	ST117/CT24	VRE	rectal swab	12-1-2017	BO233_RS09450	Chromosome
B11	ST117/CT24	VRE	rectal swab	10-1-2017	BO233_RS09450	Chromosome
B12	ST117/CT24	VRE	rectal swab	24-1-2017	BO233_RS09450	Chromosome
B13	ST117/CT24	VRE	rectal swab	12-1-2017	BO233_RS09450	Chromosome
B14	ST117/CT24	VRE	rectal swab	13-1-2017	BO233_RS09450	Chromosome
B15	ST117/CT24	VRE	rectal swab	25-1-2017	BO233_RS09450	Chromosome
B16	ST117/CT24	VRE	rectal swab	13-1-2017	BO233_RS09450	Chromosome
B17	ST117/CT24	VRE	rectal swab	26-1-2017	BO233_RS09450	Chromosome
B18	ST117/CT24	VRE	rectal swab	24-1-2017	BO233_RS09450	Chromosome
B19	ST117/CT24	VRE	rectal swab	16-1-2017	BO233_RS09450	Chromosome
B20	ST117/CT24	VRE	rectal swab	15-3-2017	BO233_RS09450	Chromosome
B21	ST117/CT24	VRE	rectal swab	13-1-2017	BO233_RS09450	Chromosome
B22	ST117/CT24	VRE	rectal swab	13-1-2017	BO233_RS09450	Chromosome
C1	ST117/CT24	VRE	rectal swab	28-10-2015	BO233_15550	Plasmid
C2	ST117/CT24	VRE	blood	18-3-2012	BO233_14905	Plasmid
C3	ST117/CT24	VRE	rectal swab	28-2-2011	BO233_14905	Plasmid
C4	ST117/CT24	VRE	rectal swab	28-2-2011	BO233_RS12430	Chromosome
C5	ST117/CT24	VRE	rectal swab	12-3-2013	BO233_15550	Plasmid
C6	ST117/CT24	VRE	blood	7-7-2012	BO233_15685	Plasmid
S1	ST117/CT24	VSE	pus	14-9-2016	NA	NA
S2	ST117/CT24	VSE	pleural fluid	9-9-2016	NA	NA
S3	ST117/CT24	VSE	rectal swab	9-9-2016	NA	NA
E1	ST117/CT24	VSE	blood	2010	NA	NA

* Insertion sites are depicted as Genbank locus_tag numbers according to the reference genome *Enterococcus faecium* E1 (Reference Sequences: NZ_CP018065.1-NZ_CP018067.1).

### Molecular Epidemiology of the Isolates

**Figure 1:**
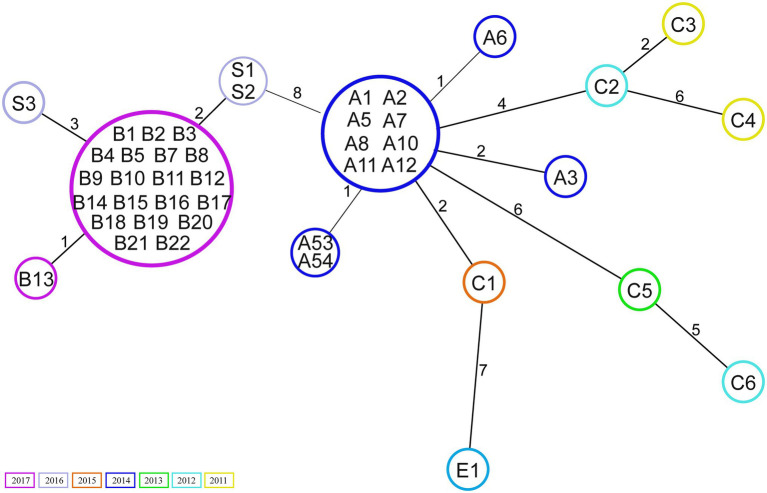
Minimum spanning tree based on core genome Multilocus sequence typing (cgMLST; 1,423 target genes). Numbers within the circle indicate the isolates. The numbers next to the lines correspond to allele differences between the isolates. Isolates are coloured by year of collection.

Core genome analysis showed that the isolates are closely related, with a minimal allelic difference lower than 10 between each cluster’ nearest isolates (Figure 1). The two major clusters correspond to outbreak events in 2014 and 2017. The former includes 10 samples previously described by Zhou et al. (2018) belonging to an outbreak (A) in April 2014. Two isolates (A53 and A54) that clustered separately were not included in the previous investigation. These were obtained from bile (A53) and blood samples (A54) of the same patient within 2weeks. Few allele differences separate this cluster from the sporadic cases collected from rectal swabs (*n*=4) or bloodstream infections (*n*=2) in different patients between 2011 and 2015. The outbreak in 2017 was presumably initiated by a patient transferred from an external hospital who was accidentally not screened for the presence of VREfm at admission nor during the stay in the previous hospital. Only after 10days, a rectal swab was collected from the patient from which a VREfm could be cultured. A subsequent screening identified 15 patients carrying an ST117/CT24 VREfm. Noteworthy, a few months before the outbreak in 2017, three VSE isolates were sequenced, two (S1 and S2) were isolated from infection sites and one (S3) from a rectal swab. These three VSE isolates turned out to be closely related to the outbreak cluster in 2017.

### Characterisation of *vanB*-Carrying Transposon *Tn*1549

The *vanB*-carrying transposon was analysed in detail using *Tn*1549/*Tn*5382 (Genbank AF192329.1) as a reference that we referred to throughout the paragraph.

First, the *vanB* operon alone was investigated by single-nucleotide polymorphism (SNP) analysis, which showed 9–10 SNP-difference between the operons of the majority of the isolates and the reference. A unique pattern was found in the operons of isolates C5 and C6. Despite being isolated in different years, they shared the same 41 SNPs. Both the nucleotide and the amino acid alignments are shown in Supplementary Figure 2.

Next, the entire sequence of the *vanB*-carrying transposon *Tn*1549 was analysed. All isolates contained the transposon. However, its genomic position was different, as indicated in detail in Table 1. The transposons of the isolates involved in the two outbreaks were characterised by the integration into the chromosome. In contrast, those of the sporadic isolates were mainly linked to plasmids except for one case (C4) in which the transposon was integrated into the chromosome. The transposon of the 12 isolates from the 2014 outbreak, 10 of which previously described by Zhou et al. (2018) was located on the bacterial chromosome, integrated into the metal-dependent phosphoesterase gene (GenBank locus tag: BO233_RS04895). The overall sequence of this transposon had a similar structure as the previously described reference transposon with 99 SNP differences.

In the 21 isolates from the 2017 outbreak, the transposon was located on the bacterial chromosome, but it was integrated upstream of the putative permease gene (GenBank locus tag: BO233_RS09450) with 98 SNP differences compared to the transposon reference sequence.

In one of the sporadic isolates collected in 2011 (C4), the transposon was integrated into the chromosome in the proximity of the *sufB* gene (GenBank locus tag: BO233_RS12430) and differed 108 SNPs from the reference sequence.

The sporadic cases of VREfm that occurred between 2011 and 2015 belonged to three separated clusters and showed significant differences: (i) in two isolates, from 2015 (C1) and 2013 (C5), the transposon integration point was located in the plasmid DNA invertase Pin gene (GenBank locus taq: BO233_15550), and their transposons differed by 101 and 260 SNPs from the reference, respectively; (ii) in one single isolate from 2012 (C6), the transposon integrated into a peptidoglycan-binding protein gene (GenBank locus taq: BO233_15685) located on the plasmid with 260 SNPs; and (iii) the *vanB* transposon found in isolates C2 (2012) and C3 (2011) was located on another plasmid and integrated upstream of the gene coding for DNA helix-turn-helix (Genbank locus taq: BO233_14905). Comparing these transposons with the reference resulted in 103 and 76 SNP differences, respectively. No IS elements were identified in the *vanB*-transposon carried by the isolates.

### Pangenome and Accessory Genome Analysis

The pangenome analysis (Supplementary Figure 1; Supplementary Table 2) revealed that 2,460 genes were present in all 43 isolates (core genes) whilst 1,433 were assigned to the accessory genome and were variably present amongst the isolates. The samples belonging to the 2017 outbreak had a different profile than the 2014 outbreak, with apparent dissimilarities in the presence/absence of accessory genes. These differences were further investigated, resulting in 362 and 216 unique genes present in 2017 and 2014 outbreak isolates, respectively. Despite half of the unique genes identified in 2017 outbreak strains encoded for hypothetical proteins, these isolates carried genes involved in mannose-specific metabolism. On the contrary, 2014 isolates had a lower proportion of genes with unknown function (*n*=73), and phage-related genes were identified in eight isolates (A5, A6, A7, A8, A10, A11, A53, and A54). A more scattered and unique pattern was observed in the single sporadic cases.

A high degree of similarity in virulence genes was observed amongst the isolates (Supplementary Figure 1). We detected in all isolates several genes encoding cell surface virulent factors involved in cell adhesion and attachment, such as *acm*, *ecbA*, *efaAfm*, *scm*, *sgrA*, and *espfm*; and pili gene cluster (PGC) proteins *pilA* and *pilB*. Amongst the secreted virulence factors that VREfm can produce, all the isolates were found to be positive for *sagA*, a major secreted antigen correlated with the binding of extracellular matrix proteins and cell growth. The *hyl* gene, a glycosyl hydrolase that belongs to the same class of secreted factors, was identified only in one of the sporadic cases isolated in 2013. Finally, in all the isolates, the gene encoding a putative transmembrane protein PTS^clin^, which phosphorylates and translocate specific carbohydrates across the cell membrane, was identified.

All VREfm carried genes associated with resistance to glycopeptides, trimethoprim (dfrG), macrolide [*erm(B)*, *mrs(C)*], and aminoglycosides [*aac(6')-Ii*, *aac(6')-aph(2'')*, *ant(6)-Ia*, *aph(2'')-Ia*, and *aph(3')-III*] (Supplementary Figure 1). These genes, except for *VanHBX*, were also detected in the vancomycin-susceptible isolates S1, S2, and S3. A gene conferring resistance to chloramphenicol (>99% of the amino acid sequence identical to *cat*, chloramphenicol O-acetyltransferase type A [EC:2.3.1.28]), was exclusively detected in the 2014 isolates and in the isolates C1, C5, and C6. Genes or chromosomal mutations associated with linezolid resistance were not detected in any of the isolates. Isolate A6 showed resistance to tigecyclin, but no gene associated with tetracycline resistance was found. Except for this isolate, the phenotypic antibiotic susceptibility patterns (Supplementary Table 3) align with the predicted resistome. Isolates C2, C3, and C6 were *vanB*-carrying VREfm with low-level resistance. All other VREfm were measured as vancomycin-resistant by the susceptibility tests.

The *in silico* analysis of the bacteriocin genes (Supplementary Figure 1) showed that enterocin A (GenBank: AAD29132.1) was detected in all isolates whilst three bacteriocins were differentially present in the investigated isolates. Lactococcin 972 (GenBank: TNX50477.1) was detected in the 2014 outbreak, C1 and C6 isolates, whilst two additional bacteriocins were present in the genome of 2017 outbreak strains and VSE isolates, namely enterocin P (GenBank: AF005726.1) and bacteriocin T8 (GenBank: DQ402539.1).

**Figure 2:**
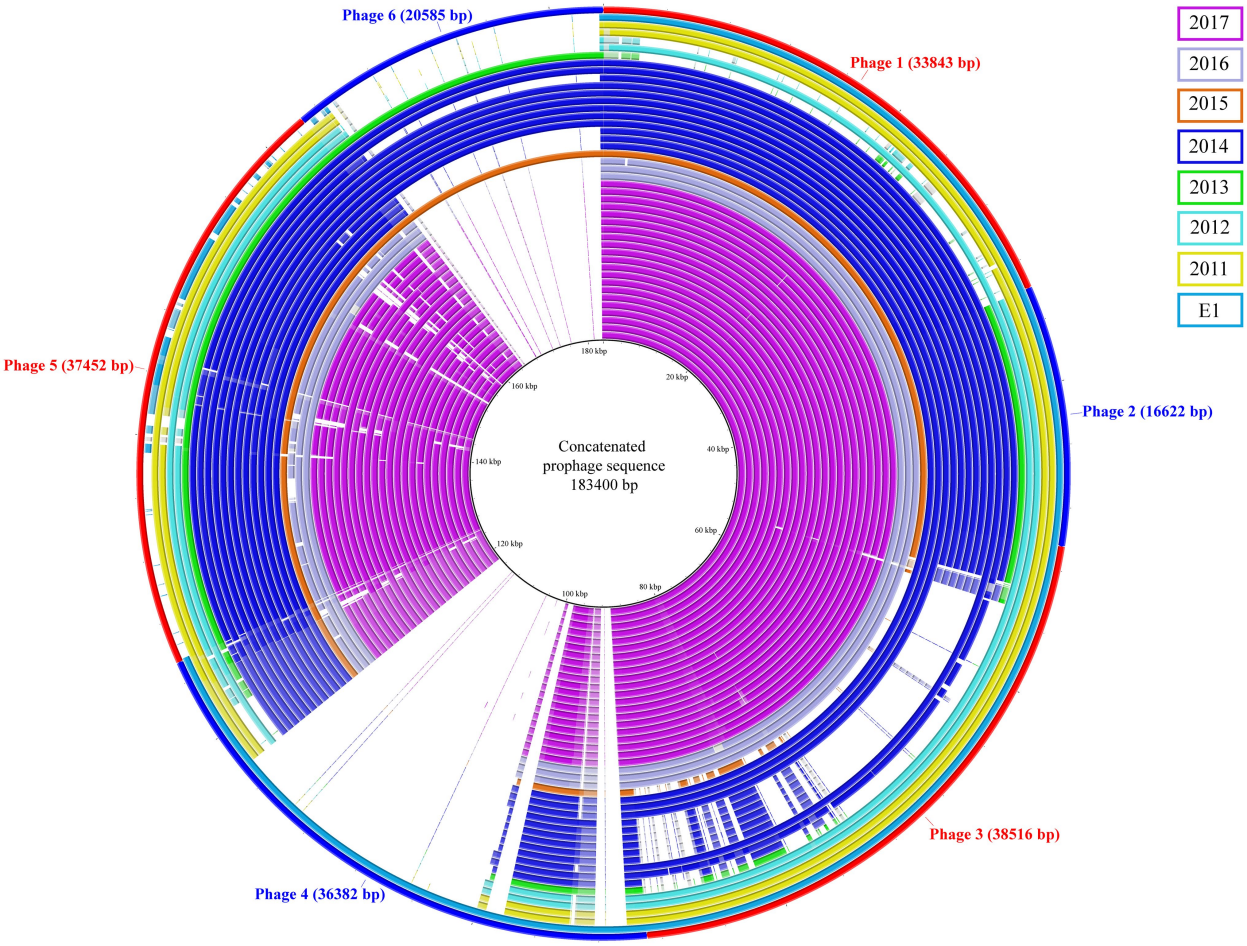
Concatenated prophage sequences identified in ST117/CT24 isolates. The concatenated reference sequence is indicated as the red-blue outer circle, and the inner circles represent the ST117/CT24 isolates coloured by the year in which they were isolated.

The prophage content of the isolates was investigated; a total of six prophages sequence were identified and used to generate a concatenated reference sequence (Figure 2). Phages 1, 2, and 5 were identified in all but two isolates (C5 and C6). A different profile was observed for phages 3 and 6: the isolates carrying phage 3 lacked phage 6 and vice versa. The former was missing in the C1, C5 and in seven isolates of the 2014 outbreak, which were instead carrying the latter; the epidemiological data analysis confirmed a possible connection between patients. Based on the bed occupancies, we observed that the patient infected with isolate A53 carried phages 3 and 6. The former was also found in 2014 outbreak isolates A1 and A12 obtained from different patients who most probably had contact. The latter phage was found in isolate A54 as well as in eight isolates of the 2014 outbreak cluster. Noteworthy isolate A54 was isolated 2weeks after A53 from the same patient, suggesting that phage 3 was lost over time. Finally, phage 4 was uniquely present in reference strain E1. Based on PHASTER analysis, the putative phages were shown to be related to phages of *Enterococcus*, *Bacillus*, and *Listeria*. However, when compared to the predicted references, the overall sequence homology was low. The highest score was observed for phage 3 that shared 17% DNA similarity with Enterococcus phage vB_EfaS_IME197 (Genbank NC_028671.2). The DNA similarity in the other phages was ranging between 3 and 11%. Detailed information on the presence of these phages and the PHASTER output is presented in Supplementary Table 4.

**Figure 3:**
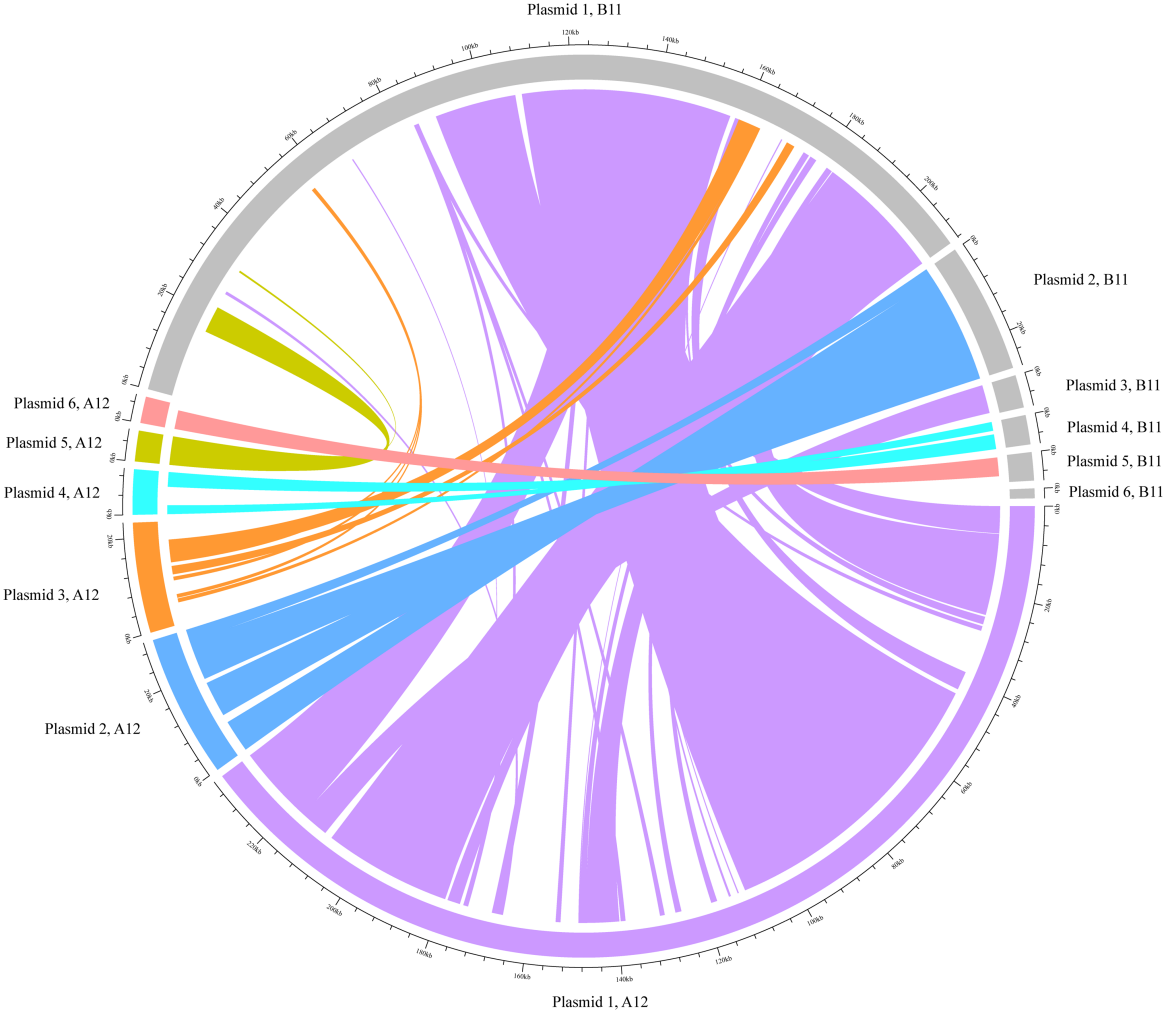
BLAST analysis of the plasmids using Circlize. The plasmid contigs from the hybrid assembles A12 and B11 were used as internal reference for the BLAST analysis. Each of A12 predicted plasmid contigs are presented in colours whilst B11 is depicted in grey.

The hybrid assemblies A12 and B11 were analysed in more detail for plasmids as they were the representative isolates of the two outbreaks in 2014 and 2017, respectively. The isolates were predicted to have each six plasmid contigs (Supplementary Table 5), which were characterised based on their RIPs and relaxase proteins. The content of these sequences was concatenated and compared. Our analysis revealed that they shared 75% of genomic similarity. Moreover, to understand if DNA rearrangements occurred in plasmids, we performed a BLAST analysis of the contigs separately (Figure 3). In both isolates, plasmid 1 was the largest (mean=223.6kpb) and contained the RepA_N initiator sequence, which showed similarity with pDO3 megaplasmid (GenBank CP003586.1). The BLAST analysis revealed a high similarity between plasmid 1 of both isolates, which shared 72% of DNA identity. One of the differences observed was the presence of enterocin P and the high-level gentamicin resistant gene [*aac(6')-aph(2'')*] in the plasmid of the B11 isolate. The second plasmid detected in isolates A12 and B11 was medium size (mean=28.9kbp) and carried a RIP protein belonging to the Inc18 family similar to pRE25 (GenBank X92945.2). This plasmid was very conserved in both isolates, which shared more than 90% similarity. In both isolates, the predicted plasmid 3 presented a variable degree of rearrangements and did not carry any RIPs. In A12, however, the gene conferring resistance to chloramphenicol was identified in this contig. The small size plasmid 4 (mean=7.8kbp) of both isolates contained a RepA protein similar to pB82 (GenBank AB178871), which belong to the Rep_3 family. The main difference between the isolates was the distinct presence of two bacteriocins: Lactococcin 972 and bacteriocin T8 were found in A12 and B11, respectively. Plasmid 6 in A12 and plasmid 5 in B11 were also classified as small plasmids (mean=5.7kbp) and shared 70% of DNA identity. They carried the RepA protein similar to pEF418 (GenBank AF408195), which belong to the Rep_3 family. Finally, plasmid 5 in A12 and plasmid 6 in B11 presented a variable degree of rearrangements and could not be assigned to any plasmid family due to the lack of replicon initiator proteins. The only relaxase family identified in the plasmids belonged to the MOB_P group. A relaxase similar to pEF1 (GenBank DQ198088.1) was found on plasmids 1 and 3 in A12 and B11, respectively. Finally, a MobA protein was found in plasmid 4 of both isolates where the closest match was with pHY (GenBank AB570326.1) and pB82 (GenBank AB178871) for A12 and B11, respectively.

## Discussion

Here, we describe the genomic makeup of 39 VREfm strains isolated from 2011 until 2017, including two consecutive outbreak isolates sharing the same genetic background and compare them with VSEfm isolates. These isolates belonged to ST117, a lineage frequently associated with nosocomial outbreaks (Papagiannitsis et al., 2017; Zhou et al., 2018; Abdelbary et al., 2019; Falgenhauer et al., 2019; Liese et al., 2019; Pinholt et al., 2019; Eisenberger et al., 2020; Weber et al., 2020) and classified as part of the hospital-associated clade A1 (Lebreton et al., 2013). A previous study showed that strains belonging to this clade have a larger genome size suggesting that the acquisition of new traits occurred whilst persisting in the hospital environment. The ability to acquire mobile elements and the flexibility to adapt under harsh conditions led to the rise of this clade (Lebreton et al., 2013). Therefore, an in-depth analysis was performed on our collection of isolates to characterise these elements and investigate if they could be used to type closely related strains belonging to ST117/CT24-*vanB* clone.

The genome analysis revealed that the isolates were closely related with only a few allele differences. According to the typing scheme proposed by de Been et al. (2015), these isolates are likely to belong to the same outbreak. In such a scenario where isolates share the same genetic background, Zhou et al. (2018) proposed to perform a detailed transposon investigation. This approach proved to increase the discriminatory power by providing additional information compared to cgMLST analysis alone (Zhou et al., 2018). Our results support this recommendation. We observed diversity in the integration point of the *Tn*1549, which suggests independent acquisitions of *vanB*-carrying transposons in investigated VREfm isolates (Howden et al., 2013; Sadowy, 2021). Moreover, by including in our analysis vancomycin-susceptible isolates as recommended in other studies (Howden et al., 2013; Bender et al., 2016; van Hal et al., 2016; Pinholt et al., 2019), we confirmed the exchange of genomic material between VSE and VRE belonging to the outbreak in 2017. Alongside, Schürch et al. (2018) also suggested the inclusion of accessory genes and plasmid analysis in addition to a SNP or gene-by-gene typing approach. Our pangenome analysis revealed that one-third of the genes could be classified as part of the accessory genome, where most of the differences amongst the isolates were noticed. The main contributors that could differentiate our isolates were plasmids, with bacteriocins and resistance genes being the main driver of these differences, and prophages.

A recent analysis of the complete plasmid sequences in a vast collection of *E. faecium* isolates (Arredondo-Alonso et al., 2020) confirmed that bacteriocins, ribosomally synthesised peptides with antibacterial activity directed against bacteria (Franz et al., 2007), were observed in hospital-associated plasmidome populations and were potentially involved in niche adaptation. Similarly, a recent study, which revealed some unpublished data on German VREfm isolates from the 1990s, classified ST117/CT24 strains as “strong producers of bacteriocins” (Werner et al., 2020). Werner et al. (2020) suggested that, through these molecules, commensal enterococci could be wiped out, leading to the expansion of this specific lineage in the gastrointestinal tract. Our data add to these findings by providing insight into the role of VSE. We observed in vancomycin-susceptible isolates the same set of bacteriocins identified in VRE isolates from the 2017 outbreak, indicating that this trait has been acquired and persisted successfully in this lineage.

The hospital-associated plasmidome populations of *E. faecium* described by Arredondo-Alonso et al. (2020) were found to carry some antibiotic resistance genes in their core structure. We observed the co-occurrence of the erythromycin resistance gene (*erm*) with aminoglycoside resistance genes in both outbreaks. Additionally, in 2014, a chloramphenicol resistance gene (*cat*) was identified in the same predicted plasmid contig with aminoglycoside resistance genes. Considering that chloramphenicol use in humans has been limited to a small number of diseases (Schwarz et al., 2004), these findings highlight the promiscuous nature that characterises hospital-associated *E. faecium* in acquiring genetic elements *via* horizontal-gene transfer (HGT; Mikalsen et al., 2015). Contrary to Arredondo-Alonso et al. (2020), our study did not identify multi-replicon plasmids; instead, single RIP families belonging to theta-replicating plasmids were detected. These findings could be explained by the limited plasmidome population investigated, which focused only on the hybrid assembly isolates.

Similar to other studies (Abdelbary et al., 2019; Falgenhauer et al., 2019; Eisenberger et al., 2020; Weber et al., 2020), we identified several virulence factors involved in surface adhesion and biofilm formation in almost all ST117 isolates investigated. Two of these genes, *pilA* and *hyl*, were carried on plasmids as described in the literature (Freitas et al., 2018; Gao et al., 2018). Our data are in line with the observation that the pattern of virulence and resistance genes herewith detected is linked to “high risk” *Enterococcus* strains (Weber et al., 2020) and is consistent with the classification within the major putative virulence markers typical of clade A1 as proposed by Freitas et al. (2018).

Although the phage analysis in this study did not allow us to clearly distinguish between closely related isolates, it revealed the constant gain and loss of genomic material that characterises *E. faecium*. For the 14 isolates collected between 2013 and 2015, we observed differential presence/absence of two prophages. However, the small set of isolates herewith included does not allow us to speculate on the stability of these elements in the enterococci genome. Despite this, previous observations suggested that bacteriophages represent the main cause of genomic diversity in *E. faecium* (van Schaik et al., 2010). One possible reason is the absence of a functional CRISPR-Cas system, which would make enterococci susceptible to phage attacks (Palmer and Gilmore, 2010; van Schaik et al., 2010). The presence of phage-like elements frequently identified in the genome of hospital-associated *E. faecium* is well documented (van Schaik et al., 2010; Mikalsen et al., 2015; Pinholt et al., 2019; Arredondo-Alonso et al., 2020). However, it is virtually impossible to classify these putative elements giving the high mosaicism that characterise their genomes (Yasmin et al., 2010; Shamia and Horsburg, 2016). Therefore, it is not surprising that the closest match identified by the web-tool PHASTER is just partially matching some elements in the predicted prophage sequences identified in the isolates of this study.

Whilst the amount of literature concerning *E. faecium* increases, less attention has been paid to understanding the mechanisms behind its ability to persist in the environment. A set of genes responsible for the survival of this opportunistic pathogen under nutrient depletion has been described in a recent study. Most of these genes were located in the core genome, where *usp*, a putative universal stress protein, was identified as a candidate that would allow the survival of *E. faecium* in PBS (de Maat et al., 2020). In our pangenome analysis, we confirmed the presence of such elements in the core genome, including in the environmental samples taken during the 2017 outbreak. Since eradicating this pathogen in hospital settings is crucial, future studies should pick up the work done by de Maat and colleagues to elucidate and further characterise the role of these genes (de Maat et al., 2020).

We acknowledge the relatively small sample size of this single-centre retrospective study, which solely focus on an individual lineage where mainly vancomycin-resistant strains have been described. Currently, in our hospital, VSEfm are not included in the sequencing surveillance, but, at the clinician’s discretion, some isolates were sequenced and have been included in this study. The lack of this data for isolates collected before 2016 does not allow us to conclude whether the outbreaks prior to this moment arose *de novo* from hospital-associated VSEfm, either by transposition of the *Tn*1549 from anaerobic gut commensals or by homologous recombination of *Tn*1549 between *vanB* VREfm and VSEfm. Despite these limitations, our study shed lights on the factors responsible for the recent expansion of specific sub-populations of VREfm within clade A1 that were not explained so far. Recently, other studies reported the spread of specific lineages throughout Europe (Bender et al., 2016; Papagiannitsis et al., 2017; Abdelbary et al., 2019; Falgenhauer et al., 2019; Liese et al., 2019; Pinholt et al., 2019; Eisenberger et al., 2020; Weber et al., 2020), amongst which ST117/CT24 has been sporadically identified carrying the *vanA* operon (Kjær Hansen et al., 2021). However, to the best of our knowledge, this is the first study reporting the molecular characterisation of the ST117/CT24-*vanB* clone and the relative contribution of its MGEs and accessory genes, therefore broadening the currently available epidemiological data.

In conclusion, our results show the importance of an extended molecular investigation that targets the core and the set of accessory genes acquired by hospital adapted strains of *E. faecium*. The analysis of the transposon and its integration point and the examination of the pangenome allowed us to better characterise VREfm clones in our hospital and conclude that identified VREfm outbreaks were caused by closely related isolates where significant differences were associated with MGEs. Thus, by employing these analyses, it is possible to gain insights into horizontal gene transfer in a selective environment such as the gastrointestinal tract of hospitalised patients.

## Data Availability Statement

The datasets presented in this study can be found in online repositories. The names of the repository/repositories and accession number(s) can be found at: https://www.ebi.ac.uk/ena (PRJEB42347, PRJEB41626, and PRJEB25590) and https://www.ncbi.nlm.nih.gov/ (PRJNA725797).

## Author Contributions

MC-F, JR, XZ, AF, and EB conceived the study. PL performed the formal analysis and visualisation of the data and wrote the manuscript. NC analysed the data and revised the manuscript. SR analysed the data. ML was responsible for the clinical data. XZ and EB revised the manuscript and commented on the interpretation of the results. HH, JR, and AF contributed to the interpretation of data and revised the manuscript. MC-F contributed to the analysis and supervised the study throughout. All authors contributed to the article and approved the submitted version.

## Funding

This study was supported by the Marie Skłodowska-Curie Actions (Grant Agreement number: 713660 – PRONKJEWAIL – H2020-MSCA-COFUND-2015).

## Conflict of Interest

JR is currently employed by IDbyDNA Inc.

The remaining authors declare that the research was conducted in the absence of any commercial or financial relationships that could be construed as a potential conflict of interest.

## Publisher’s Note

All claims expressed in this article are solely those of the authors and do not necessarily represent those of their affiliated organizations, or those of the publisher, the editors and the reviewers. Any product that may be evaluated in this article, or claim that may be made by its manufacturer, is not guaranteed or endorsed by the publisher.

## Supplementary Material

Supplementary Figure 1 Overview of the pangenome and accessory genes analysis. The neighbour-joining tree is based on cgMLST (1,423 target genes). The presence and absence of the predicted resistances genes, bacteriocin, and virulence factors are indicated with coloured and white cells, respectively. Similarly, the presence and absence of a gene in the pangenome matrix is depicted with blue and white lines, respectively. On the pangenome matrix, the *in silico* predicted bacteriocins and resistance genes which could distinguish the clusters have been highlighted with orange and red lines, respectively.

Supplementary Figure 2 Nucleotide and amino acid alignment of vanB gene in the representative isolates. Clonal isolates (100% identity) from 2014 and 2017 outbreaks are not depicted in the alignments and were represented by isolates A12 and B11, respectively.

Supplementary Table 1 Assembly statistics.

Supplementary Table 2 Pangenome analysis results. List of all the genes identified in the isolates and those uniquely reported in the 2014 and 2017 outbreak.

Supplementary Table 3 Summary of the phenotypic antibiotic susceptibility test in the isolates included in the study.

Supplementary Table 4 Summary of the putative phages identified in this study. The presence and absence of the phages have been indicated with 1 and 0, respectively. The output generated by PHASTER is ordered according to the phage ID reported in the study.

Supplementary Table 5 mlplasmid results of the hybrid assemblies A12 and B11. The predicted plasmid contigs have been renamed in ascending order. Detailed information concerning the RIP families and relaxase protein is herewith summarised.
