# Supplementary figures and images for: Molecular Characterisation of Vancomycin-Resistant Enterococcus faecium Isolates Belonging to the Lineage ST117/CT24 Causing Hospital Outbreaks
Source: Front Microbiol. 2021 Sep 27;12:728356. doi: 10.3389/fmicb.2021.728356 (PMC8503688; doi:10.3389/fmicb.2021.728356)

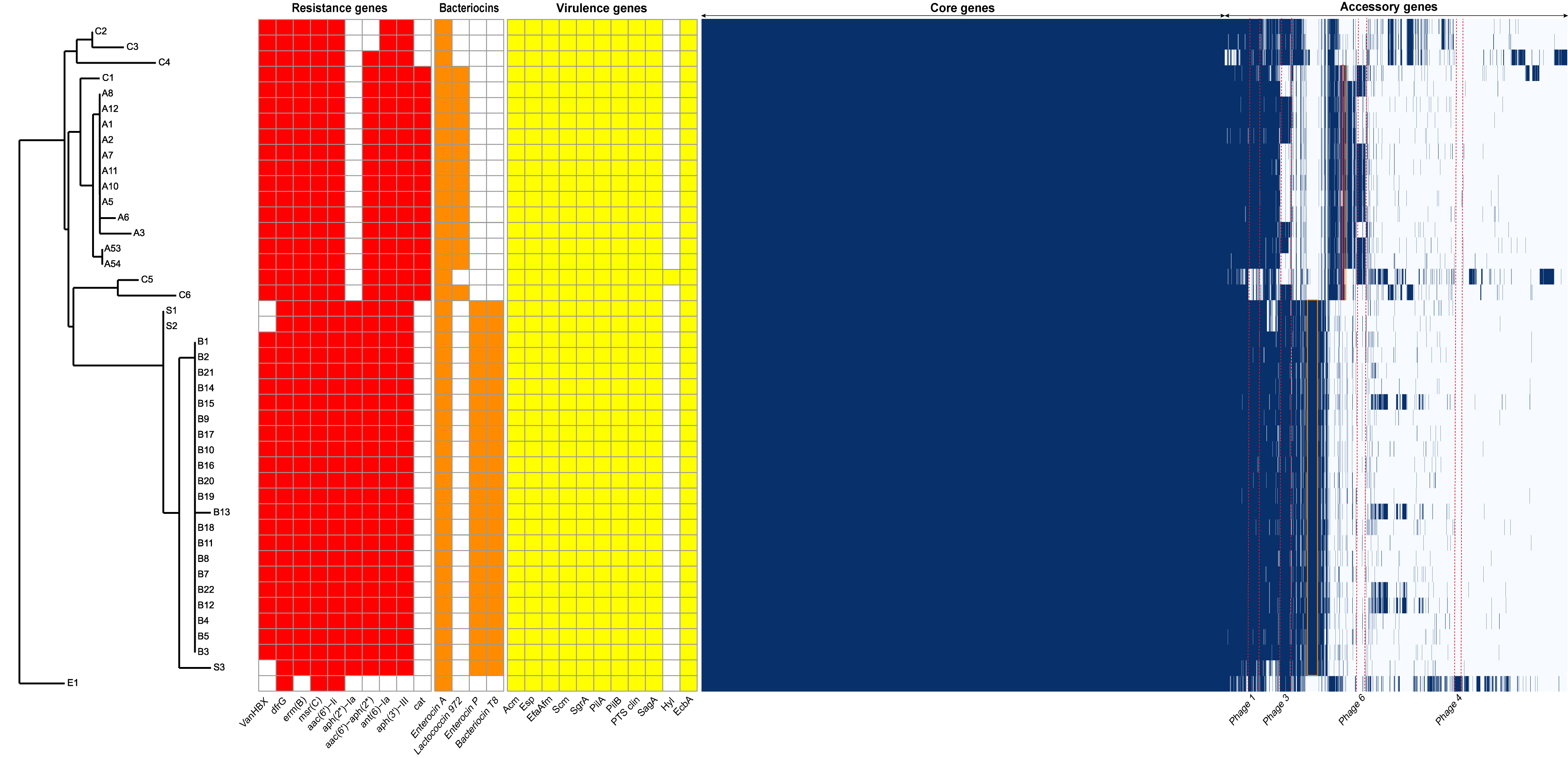

Supplement: Supplementary Figure 1 — Overview of the pangenome and accessory genes analysis. The neighbour-joining tree is based on cgMLST (1,423 target genes). The presence and absence of the predicted resistances genes, bacteriocin, and virulence factors are indicated with coloured and white cells, respectively. Similarly, the presence and absence of a gene in the pangenome matrix is depicted with blue and white lines, respectively. On the pangenome matrix, the in silico predicted bacteriocins and resistance genes which could distinguish the clusters have been highlighted with orange and red lines, respectively. [file Image_1.TIF]

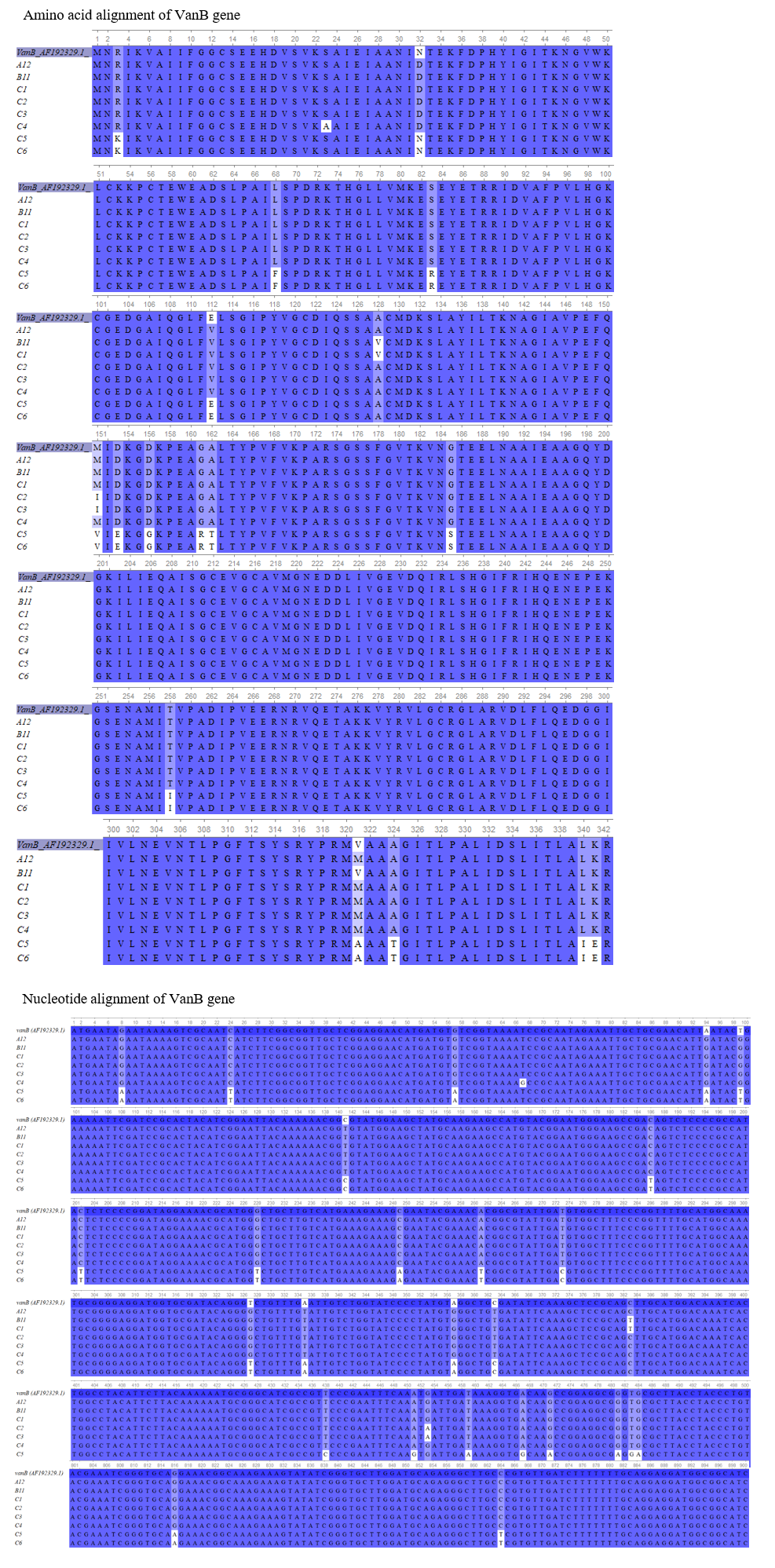

Supplement: Supplementary Figure 2 — Nucleotide and amino acid alignment of vanB gene in the representative isolates. Clonal isolates (100% identity) from 2014 and 2017 outbreaks are not depicted in the alignments and were represented by isolates A12 and B11, respectively. [file Image_2.TIF]
